# Sharing a Context with Other Rewarding Events Increases the Probability that Neutral Events will be Recollected

**DOI:** 10.3389/fnhum.2015.00683

**Published:** 2016-01-08

**Authors:** Eleanor Loh, Matthew Deacon, Lieke de Boer, Raymond J. Dolan, Emrah Duzel

**Affiliations:** ^1^Wellcome Trust Centre for Neuroimaging, University College LondonLondon, UK; ^2^Institute of Cognitive Neuroscience, University College LondonLondon, UK; ^3^Berlin School of Mind and Brain, Humboldt UniversityBerlin, Germany; ^4^Max Planck UCL Centre for Computational Psychiatry and Ageing Research, University College LondonLondon, UK; ^5^Institute of Cognitive Neurology and Dementia Research, Otto-von-Guericke UniversityMagdeburg, Germany

**Keywords:** context, memory, recollection, reward, hippocampus, dopamine

## Abstract

Although reward is known to enhance memory for reward-predicting events, the extent to which such memory effects spread to associated (neutral) events is unclear. Using a between-subject design, we examined how sharing a background context with rewarding events influenced memory for motivationally neutral events (tested after a 5 days delay). We found that sharing a visually rich context with rewarding objects during encoding increased the probability that neutral objects would be successfully recollected during memory test, as opposed to merely being recognized without any recall of associative detail. In contrast, such an effect was not seen when the context was not explicitly demarcated and objects were presented against a blank black background. These qualitative changes in memory were observed in the absence of any effects on overall recognition (as measured by d′). Additionally, a follow-up study failed to find any evidence to suggest that the mere presence of a context picture in the background during encoding (i.e., without the reward manipulation) produced any such qualitative changes in memory. These results suggest that reward enhances recollection for rewarding objects as well as other non-rewarding events that are representationally linked to the same context.

## Introduction

Reward associations are known to enhance memory for the reward-predicting event (Wittmann et al., 2005; Adcock et al., 2006), a phenomenon that has been linked to a reward-related activation of the hippocampus and the substantia nigra/ventral tegmental area (see Lisman et al., 2011, for review). Dopamine released from the substantia nigra/ventral tegmental area is thought to bring about such mnemonic benefits by stabilizing synaptic plasticity in hippocampal neurons, thus rendering newly formed memories long-lasting (Frey and Morris, 1997; Bethus et al., 2010; Chowdhury et al., 2012; for review see Shohamy and Adcock, 2010; Lisman et al., 2011; and Redondo and Morris, 2011). In addition to improving memory for reward-predicting events, dopamine is thought to additionally stabilize memory traces for *neutral* events that occur within the same temporal window, because the resultant availability of plasticity related proteins allows for stabilization of synaptic changes of these neutral events as well (a phenomena known as synaptic tag-and-capture; see Redondo and Morris, 2011, for review).

Although the synaptic tag-and capture hypothesis predicts such cross-stimulus memory enhancement, the evidence for such an effect in humans is unclear. While some researchers have found improved memory for neutral stimuli that are presented immediately prior to the reward-predicting event (Murayama and Kitagami, 2014), others have found that reward fails to improve memory for neutral stimuli that are presented in close temporal proximity to other reward-predicting ones (Wittmann et al., 2011). One possibility is that the extent of cross-stimulus memory enhancement may rely on the associative links between the rewarding and neutral event, rather than strict temporal co-occurrence. Consistent with this hypothesis, reward has been shown to selectively benefit memory for *neutral* stimuli that are in the same semantic category (e.g., fish) as other rewarded stimuli, despite presentation of the rewarding and neutral stimuli being temporally spread out over the course of the experimental session (Imai et al., 2014). Similar effects have been demonstrated in the domain of decision making, wherein choices between two neutral options is influenced by each options’ indirect (i.e., via other intervening stimuli) associations with reward (Wimmer and Shohamy, 2012). As such, neutral objects that are embedded within the same background context as reward-predicting ones may thus be linked at the level of the hippocampal ensemble, so that mechanisms that lead to improved memory of the rewarding event (e.g., enhanced consolidation) inadvertently lead to enhanced memory for the entire mnemonic ensemble.

In this study, we set out to examine if memory for neutral events was improved by having shared a visually rich background context with separate rewarded events (versus being presented against a blank black background). Additionally, we examined if the similarity of the background context had any effect on memory or any context-mediated effects of reward. We hypothesized that the presence of a shared background picture would improve memory for neutral objects that were embedded in the same context as rewarding ones, and that the similarity of the background context might further modulate such context-mediated effects, given the demonstrated necessity of the hippocampus in supporting reliable disambiguation of similar contexts that cannot be disambiguated on the basis of single features (Graham et al., 2006; McHugh et al., 2007; Neunuebel and Knierim, 2014).

**FIGURE 1:**
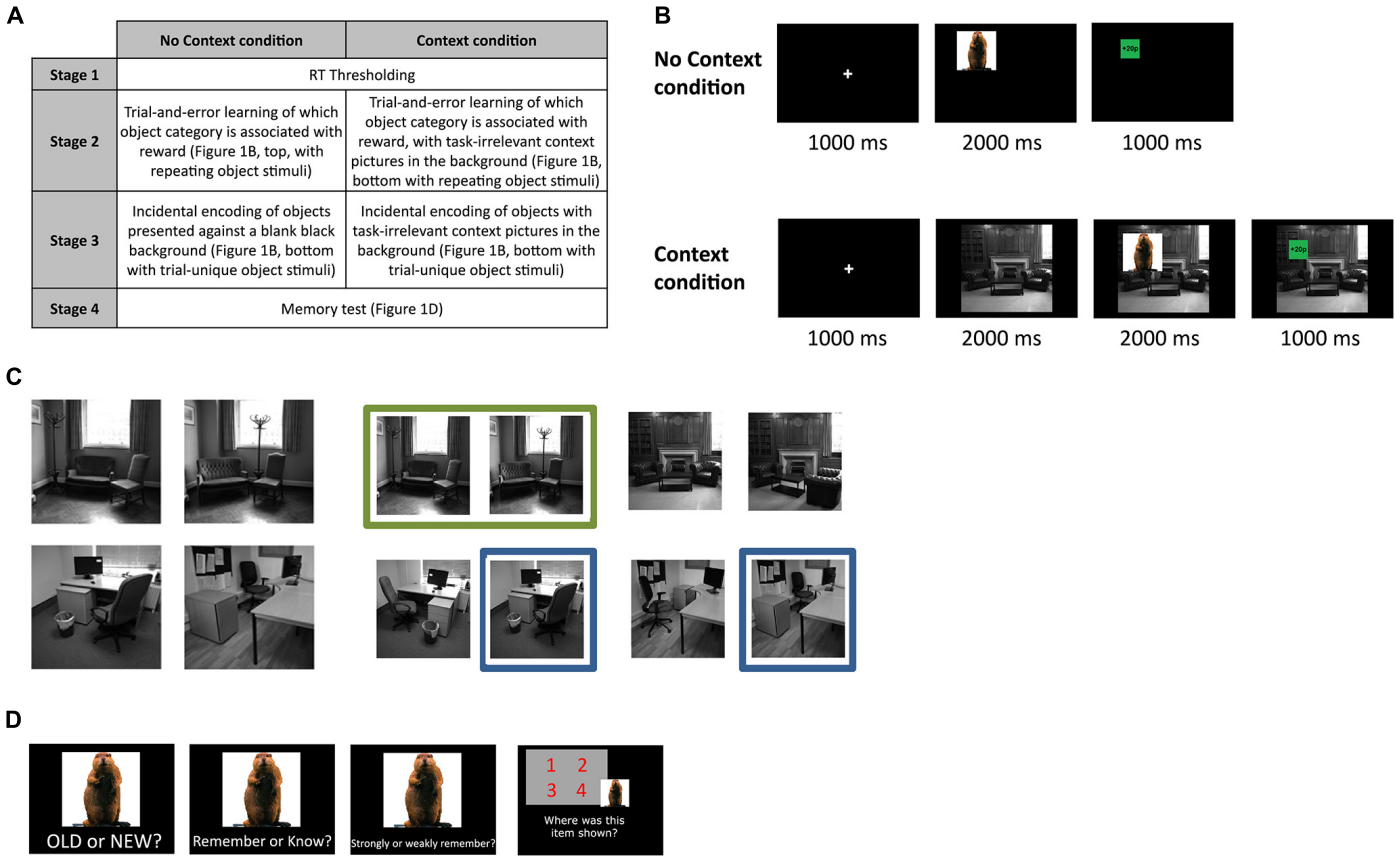
**Experimental design. (A)** Experimental sessions for subjects in each condition **(B)** Trial sequences for the reward-learning and encoding stage, for the no context and context condition in Experiment 1 (top and bottom, respectively). **(C)** Context pictures used in the context condition. Each subject saw four unique context pictures (left), which consisted of one similar and one dissimilar picture pair (see Materials and Methods for more detail). To eliminate any effects that related to specific context stimuli themselves, we counterbalanced the exact context pictures used for each individual subject, by drawing the four stimuli pseudo-randomly from the overall pool of context pictures (right). **(D)** Five days later, subjects returned to the lab for a surprise memory test (procedure identical for all subjects).

Two groups of subjects made semantic judgments to trial-unique object pictures, where the semantic category of the object (man-made or natural) indicated whether they were able to win money on that trial or not (Experiment 1). In one group (context condition; **Figure 1B**, bottom), these objects were presented against a backdrop of repeating context pictures (two similar, two dissimilar). In the other group (no context condition; **Figure 1B**, top), the objects were presented alone against a blank black background without any explicit background context. In addition to analyzing different memory measures independently, we also directly compared remember and know type memories to see if our experimental manipulation had any effect on the quality of subsequent memory. Lastly, we conducted a second follow-up study (Experiment 2) in order to control for the possibility that the mere presentation of a context picture (i.e., without any reward manipulation) may itself have influenced subsequent memory.

## Experiment 1: Materials and Methods

### Subjects

Thirty-two subjects (19 female, mean age = 23.13, *SD* = 3.21) participated in Experiment 1, randomly divided across the context and no context condition (i.e., between-subject design). Two subjects were excluded from analysis because they misunderstood instructions for the memory test, producing *n* = 14 and *n* = 16 for the context and no context conditions, respectively. All subjects were recruited from the local population via departmental subject recruitment pools, had normal or corrected-to-normal visual acuity, and reported no history of neurological or psychiatric conditions, or significant medications. All experiments were run with each subject’s written informed consent and according to the local ethics clearance (University College London, London, UK). Subjects were compensated for their time at a rate of £6/h, plus additional money to be won on the task itself.

### Experimental Procedure

The experiment included four separate stages that were completed by all subjects in both conditions (see **Figure 1A** for overview).

In stage 1 (thresholding), we determined individual reaction time thesholds for category decisions. Subjects made speeded responses to 30 object pictures, specifying whether each object was man-made or natural (i.e., non-man-made; 15 objects in each category). Each object was repeated twice, to make a total of 60 trials in this stage. Response times (RTs) from this thresholding task were used to determine the threshold for rapid responses in all other stages of the experiment, calculated as the mean plus the standard deviation of all RTs where the subjects responded correctly in the semantic categorization task.

The second and third stages of the experiment (reward-learning and encoding stages, respectively) differed for subjects in the context versus no context conditions. In the second stage of the experiment (reward-learning stage), all subjects learned via trial and error which object category was associated with reward, and which category was not. The particular semantic category that was associated with reward was counterbalanced across all subjects. For all subjects, the object semantic category probabilistically predicted reward availability (with a 1/8 chance of a category-incongruent outcome).

In Stage 2, subjects in the no context condition viewed a fixation cross (1000 ms), followed by an object picture presented for 2000 ms in one of the four quadrants onscreen (**Figure 1B**, top). Like in the previous stage, subjects made speeded semantic categorization responses to each object, and then were given feedback regarding whether they had won money or not, or whether their response had been inaccurate or too slow. Subjects were told that one of the object categories was to be associated with reward, while the other was not. If the object category indicated an availability of reward on that trial, they would then win that reward by being fast and accurate in the semantic judgments made to the objects. Subjects in the context condition viewed trials that were similar to that in the no context condition, except that an additional context stimulus was briefly presented by itself for 2000 ms after the fixation cross, and stayed onscreen for the rest of the trial, with the object stimulus and outcome presented on top of this context picture that remained in the background (**Figure 1B**, bottom). Subjects were told to ignore the context stimuli as there was no relationship between the context picture and reward, or anything else in the task. All subjects completed 64 trials of their respective tasks, involving 20 object stimuli (each repeated approximately three times, not subject to memory test) and four context pictures (for the context condition only; **Figure 1C**, left; each picture repeated 16 times). In addition to having subjects learn which object category went with reward prior to encoding (i.e., the next stage of the experiment), this initial reward-learning phase also served to pre-expose subjects to the context stimuli, so as to prevent the occurrence of contextual *novelty* effects on memory. Subjects were told to respond during every single trial, regardless of whether there was money to be won or not, and were also instructed that a proportion of the total amount of money that they won during the entire experiment would be paid to them in addition to the money that they would receive as compensation for the time spent. Verbal report confirmed that all subjects had accurately determined which object category was associated with reward, by the end of this session.

Stage 3 of the experiment (encoding) was identical to the second stage (**Figure 1B**), except that the object stimuli presented during this stage of the experiment were entirely trial-unique. Like before, subjects in the no context condition saw objects onscreen without any context stimuli shown in the background, while subjects in the context condition saw context pictures first presented alone, and then with objects overlaid on top of them. None of the object stimuli from the previous two stages were repeated during this or any other stages of the experiment. Two hundred forty trials of this encoding stage were completed. Subjects were not explicitly told that their memory for the objects would be tested later, but were instead instructed to continue performing the task to the best of their ability as they had in the previous stage.

During Stage 4 of the experiment (memory test, **Figure 1D**; identical for both the context and no context conditions), conducted 5 days later, subjects then saw 360 object pictures on a computer screen (240 of which they had seen before in Stage 3 of the experiment, 120 of which were new), and for each object had to decide whether it was old (i.e., if they had seen it before in the experiment) or new. If the object was deemed to be old, subjects were then asked if they “knew” or “remembered” the object. Following this judgment, subjects were asked to indicate how confident their memory for the object was by stating whether it was “strong” or “weak”. We followed standard procedures in instructing subjects about remember and know judgments (Tulving, 1985); specifically, subjects were instructed to give a ‘remember’ response if they could recollect any other details from when they had initially seen the object, and were instructed to respond with ‘know’ if they could not recollect any other such detail and merely had a sense of it being familiar. Detailed instructions regarding this distinction were relayed to subjects, along with examples of each memory type as one would encounter them in daily life, to ensure that subjects understood how they should respond in the task. Because ‘remember’ and ‘know’ responses are mutually exclusive in our memory test procedures, this experiment should be thought of as comparing successful recollection (in the case of ‘remembered’ objects) with failed recollection in the face of successful familiarity-based recognition (in the case of ‘known’ objects). Lastly, subjects then had to indicate which quadrant of the screen they had previously seen the object in (position recall), if they had earlier indicated that object to be old. All responses in this stage of the experiment were self-paced.

### Stimuli and Design

Object stimuli consisted of color images assembled from a database of object stimuli (Brady et al., 2008), as well as some additional images from the internet, and were balanced in terms of semantic category (man-made versus natural). The context stimuli used in the Context condition consisted of grayscale pictures of offices and living rooms with no human beings in them. Each subject saw 4 unique context stimuli, repeated randomly over the course of the experiment, which consisted of a ‘similar’ context pair and a ‘dissimilar’ pair. The similar context stimuli were pictures of the same room wherein the position of the furniture had been noticeably altered (without adding or removing any elements in the scene), while the dissimilar context stimuli consisted of two pictures of entirely different rooms that belonged to the same semantic category (office or living room). The exact context stimuli used for each subject was counterbalanced across the entire group, by creating a pool of four similar context-picture pairs (two living room and two office; **Figure 1C**, right), and drawing different similar-dissimilar permutations of the context stimuli from the original four similar context-pairs for each subject (**Figure 1C**). The similar and dissimilar context pairs were included in this study to enable us to determine if the context similarity (and implicit pattern-separation load involved in discrimination) had any influence on the memory effects anticipated.

### Behavioral Measures and Analyses

Accuracy scores and RTs of the semantic judgments made during the encoding stage were analyzed with a mixed 2 × 2 ANOVA (context condition × valence; context condition, between-subject: context versus no context; valence, within-subject: reward vs. neutral), to verify good learning of the object-category and reward associations. All memory measures (e.g., remember rate) were corrected for false alarms, except for position recall accuracy, which was calculated as the number of objects for which position was correctly recalled, divided by the number of objects that were recognized to be old. Position recall accuracy was computed in this way so as to compensate for the fact that memory for the position was only tested on trials in which subjects had recognized the object to be old. To compare different types of memories, remember and know rates were analyzed with a 2 × 2 × 2 (memory type × context condition × valence) ANOVA. Associative memory and d’ memory scores were also analyzed individually with a mixed 2 × 2 (context condition × valence) ANOVA (same factors as with the encoding-stage data).

To examine the effect of context similarity and object reward on performance, we analyzed data from the context condition only with a repeated measures 2 × 2 ANOVA (similarity: similar vs. dissimilar; valence: reward vs. neutral). Encoding-stage accuracy scores, RTs and all memory scores were analyzed using this same procedure.

## Experiment 1: Results

### Encoding-Stage Performance

**FIGURE 2:**
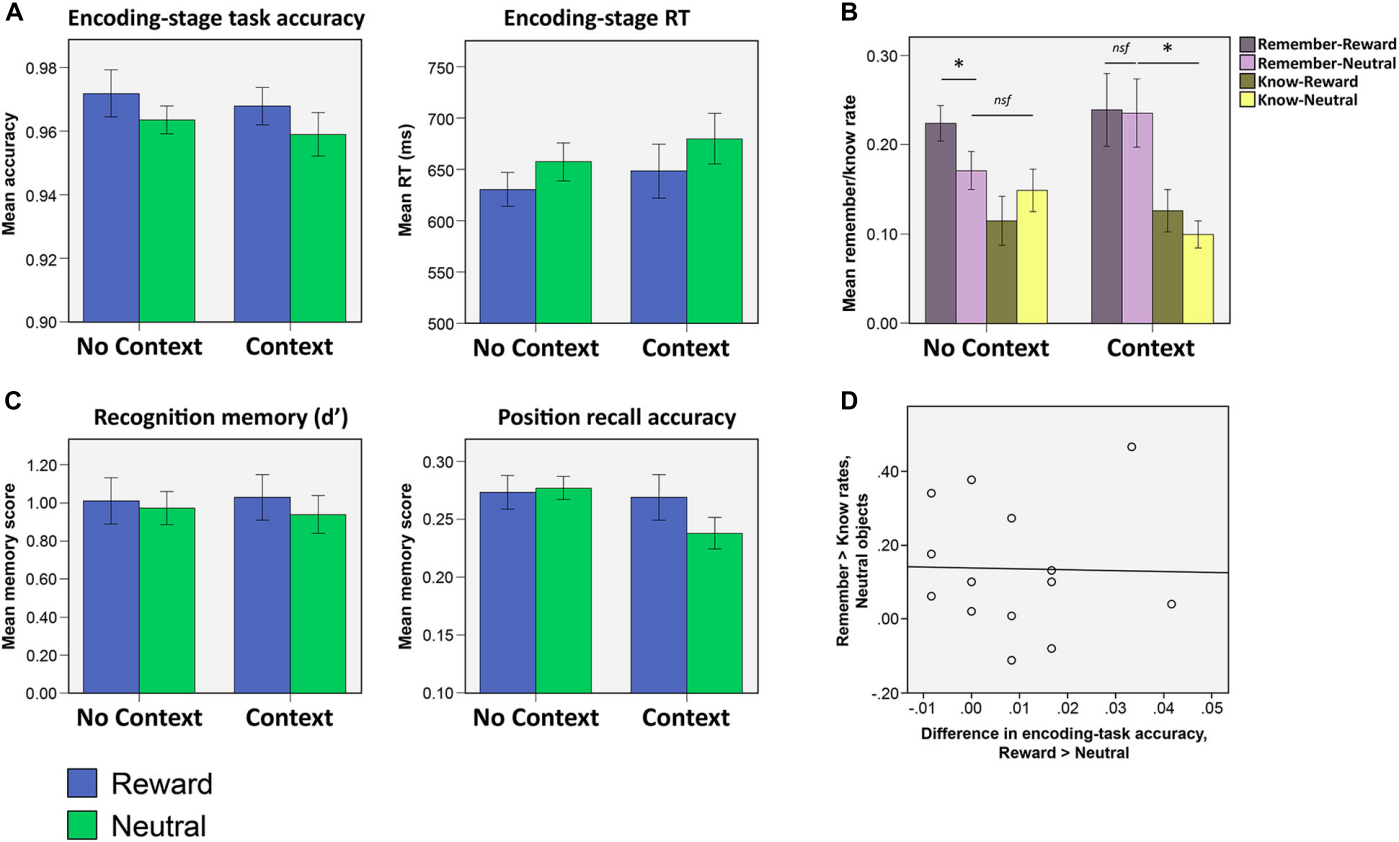
**Results from Experiment 1. (A)** Encoding-stage task accuracy and response times (RTs). Reward significantly influenced by task accuracy and RTs, but context condition did not significantly influence either of these measures, either alone or in interaction with reward. **(B)** Recognition and position recall accuracy after a 5 days delay. Neither reward nor context significantly influenced either of these memory measures (asterisks indicate significant differences at the level *p* < 0.05). **(C)** Rates of ‘remember’ and ‘know’ responses for recognized objects. In the no context condition, subjects reported higher rates of ‘remembering’ for rewarded objects as compared to neutral. In the context condition, however, both neutral and rewarded objects were more likely to be recognized on the basis of recollection (as opposed to merely being recognized without any recall of associative detail). **(D)** In the context condition, encoding-stage task accuracy was not correlated (across all subjects) with the difference in remember rates for the neutral versus the rewarding objects.

All subjects accurately reported which object category had been associated with reward, after the reward-learning stage. When subjects in the context condition were explicitly asked if they had observed any relationship between the background pictures and the objects’ reward status, none of the subjects reported having noticed any such relationship. Consistently, subjects in both conditions were quicker to respond for objects where reward was available, compared to objects where reward was not [**Figure 2A**; Main effect of valence, *F*(1,28) = 11.05, *p* = 0.002]. No main effect of context condition or valence by context condition interaction was observed in the RT data (both *p* > 0.4). Overall, mean accuracy of semantic judgments was high (mean accuracy = 0.98, *SD* = 0.04), and though no main effect of context condition or condition by valence interaction was observed (both *p* > 0.5), a main effect of reward was observed [*F*(1,28) = 4.42, *p* = 0.045], with higher accuracy in the reward condition as compared to the neutral one (**Figure 2A**). This main effect of valence opens up the possibility that subjects may have been paying more attention to the objects when they were rewarding as opposed to when they were not. This effect is surprising, however, given that subjects would have had to process the objects semantically in order to discern their reward status (having not received any preceding cues to indicate the reward status of the upcoming object that might have enabled them to disengage attentionally). As such, it seems likely that subjects may have been more prone respond with the rewarding object-category, since this response involved potential gain with no penalty for incorrect responses. Nevertheless, to mitigate the effects that such attentional errors may have on the memory results, all memory scores were calculated by excluding any objects that had received an incorrect response during the encoding stage.

### Sharing a Context with Rewarded Objects Increases the Probability that Neutral Objects will be Recollected as Opposed to being Recognized Without any Recall of Associative Detail

When analyzed separately, none of the memory scores (d′, remember, know, sure-remember rates, sure-know rates, or position recall accuracy) showed any significant effects of object valence, the context condition, or by the interaction between the two (d′ and position recall accuracy shown in **Figure 2B**; all main effects and interaction, *p* > 0.094). Comparing remember and know rates with a mixed 2 × 2 × 2 (memory type x context condition x valence) ANOVA, however, revealed both a main effect of memory type [*F*(1,28) = 11.30, *p* = 0.002] which reflected higher rates of ‘remember’ compared to ‘know’ memories, as well as a three-way interaction between memory type, context condition and valence [*F*(1,28) = 6.79, *p* = 0.015].

To clarify the nature of the three-way interaction, 2 × 2 (memory type × object valence) ANOVAs were run separately on remember and know rates in each context condition (**Figure 2C**; all statistics listed in **Table 1**). This analysis indicated that the three-way interaction was driven by high rates of remembering for rewarding objects in the no context condition, and high rates of remembering for rewarding as well as *neutral* objects in the context condition. The three-way interaction was driven by the presence of a memory type × valence interaction in the no context condition, which was driven by greater remembering of rewarded objects and an absence of any such valence effect on know rates [remember: *t*(15) = 2.71, *p* = 0.016; know: *p* > 0.2]. In the context condition, no such memory type × valence interaction was observed (*p* > 0.4). Instead, remember rates were significantly higher than know rates, for both rewarded and neutral objects (i.e., main effect of memory type, **Table 2**). No significant differences were found comparing the remember rates for the rewarded and neutral objects in the context condition to the remember rates for rewarded objects in the no context condition (all *p* > 0.7).

**Table 1:** Memory type and valence effects within the three way interaction.

	No context condition	Context condition
Memory type	*F*(1,15) = 4.03, *p* = 0.063	*F*(1,13) = 6.65, *p* = 0.021^∗^
Valence	*F*(1,15) = 0.24, *p* > 0.6	*F*(1,13) = 0.55, *p* > 0.4
Memory type × valence	*F*(1,15) = 8.80, *p* = 0.01^∗^	*F*(1,13) = 0.58, *p* > 0.4

**Table 2:** Experiment 2 memory statistics.

	*t*-statistic (*df* = 21)	*P*-value (2-tailed)
Recognition (d′)	0.70	0.49
Sure hit rate	1.60	0.13
Remember rate	0.63	0.54 (0.27, one-tailed)
Know rate	1.60	0.12
Sure remember rate	1.15	0.26
Sure know rate	1.43	0.17
Position recall accuracy	0.71	0.49

Comparing remember rates alone across context and no context conditions failed to clarify the nature of the interaction, in that direct comparison of remember rates for neutral objects in the context vs. no context condition failed to find a significant effect (*p* > 0.1). Focusing on the *difference* in the rates of ‘remember‘ and ‘know’ responses to neutral objects (i.e., remember > know rates for neutral objects), however, we found that neutral objects were more likely to be remembered rather than known to be old, in the context condition [*t*(13) = 2.98, *p* = 0.011] but not the no context condition (*p* > 0.5). As such, we find that neutral objects more likely to be remembered than known to be old (i.e., similar to rewarding objects), in the context condition, whereas memory for the neutral objects in the no context condition did not show such a pattern to any significant extent. These results indicate that reward associations modulate the quality of memories in the absence of explicit context stimuli. However, inclusion of an explicit (but task-irrelevant) context stimulus leads to better remembering of associative detail (resulting in a ‘remember’ rather than a ‘know’ response) that benefits rewarded as well as neutral objects that are encountered within the same repeating context.

### Remembering of Neutral Objects in the Context Condition was not Related to Attentional Disengagement During Encoding

We noted earlier that subjects were more likely to make incorrect responses during the encoding task when the objects were neutral as compared to rewarded (**Figure 2A**). An additional possibility that we wanted to explore was the possibility that the observed pattern of memory results (i.e., greater likelihood of remembering of neutral objects in the context condition) may have come about as a result of such attentional disengagement from neutral object processing: it is conceivable, for example, that attentional disengagement in the context condition would have led subjects to pay greater attention to the context stimuli, leading to a greater propensity to report such objects as being remembered due to stronger incidental encoding of the context stimulus in the background. If this had been the case, we reasoned that greater attentional disengagement (indexed by a greater reward-related difference in encoding-stage accuracy scores) might be related to the observed enhancement of remembering for neutral objects in the context condition. To test for this, we looked to see if reward-related differences in encoding task accuracy were correlated, across all subjects, with reward-related differences in remember rates. No such correlation was observed (**Figure 2D**; *r* = -0.15, *p* > 0.6). As such, we found no evidence to suggest that the observed remembering of neutral objects was due to subjects in this condition having shifted their attention from the object to the context stimuli (i.e., leading to both poorer encoding task accuracy and a greater propensity for such objects to be remembered due to good recall of the context pictures).

### Context Similarity did not have any Effect on Memory Measures

**FIGURE 3:**
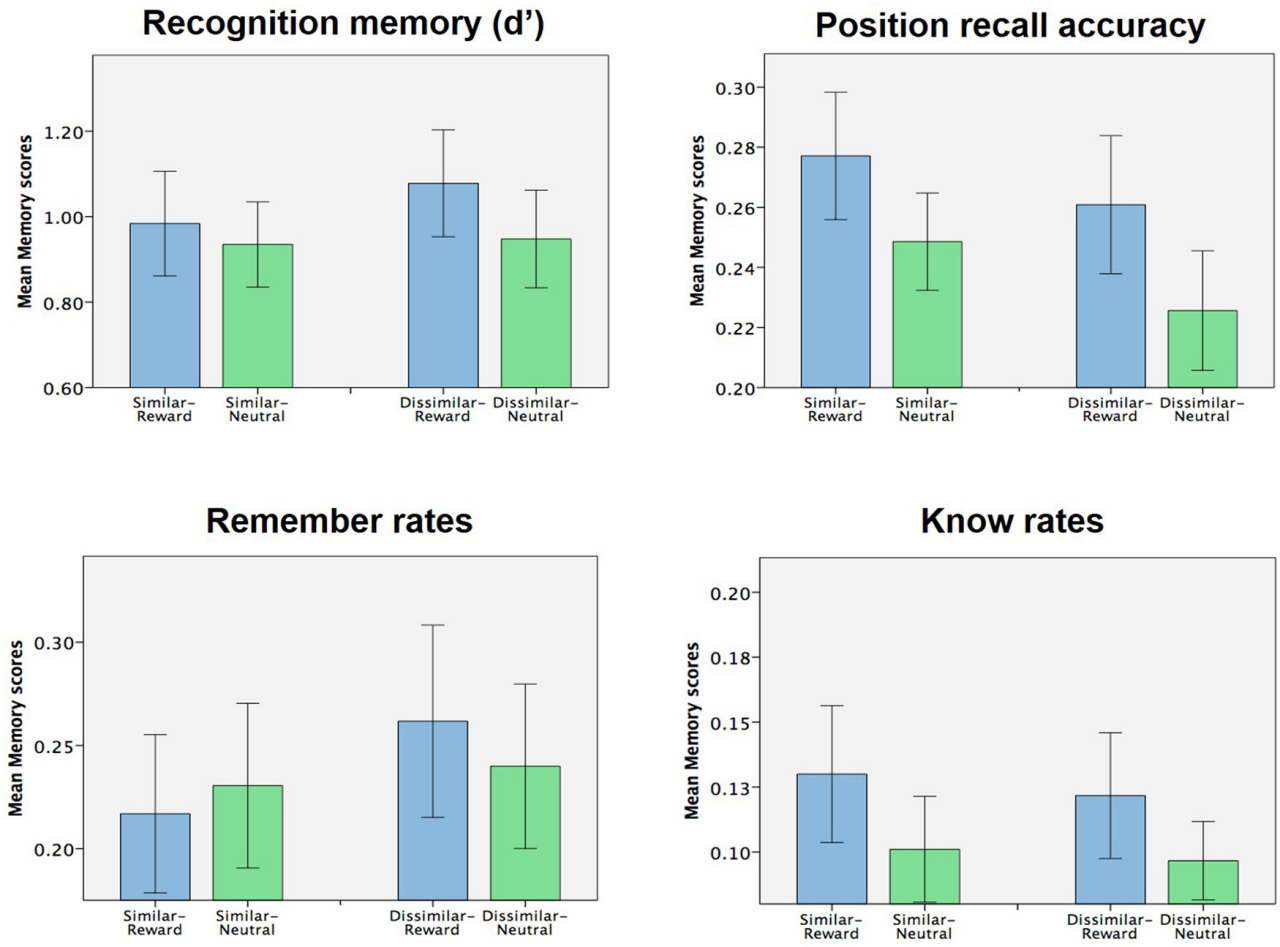
**Effect of context similarity of memory (Experiment 1).** No effects of context similarity (nor interaction of context similarity with object valence) were observed for any of the memory measures collected.

Our experimental design also allowed us to examine if context similarity had any effect on subsequent memory. Focusing solely on the context condition, we analyzed encoding-stage task accuracy, RTs, and all memory measures with a 2 × 2 ANOVA (similarity × valence). Aside from the already-noted effects of valence on (encoding-stage) task accuracy and RT, no other main effects of similarity or interactions between similarity and valence were found in encoding-stage behavioral measures (all *p* > 0.1). Additionally, no main or interacting effects were observed on any of the memory measures tested (d′, position recall accuracy, remember and know rates; **Figure 3**, all *p* > 0.1). As such, we failed to find any evidence that context similarity influenced memory or any of the previously reported memory effects, either on its own or in interaction with the effects of object reward.

### Alternative Explanations to be Controlled for

The results of Experiment 1 indicated that sharing a context with a rewarded object qualitatively changed memory for *neutral* objects, without necessarily improving overall recognition. An alternative explanation that we were unable to control for within the same experiment is that the presence of an explicit context picture might *per se* lead to a greater probability of recollection-based recognition, even in the absence of any reward manipulation. Such an explanation is highly plausible, given that awareness of associated contextual detail is a hallmark characteristic of ‘remember’ memories, and is indeed the criteria with which subjects were instructed to base the remember/know distinctions on (Tulving, 1985). As such, we decided to explicitly examine this issue by running a separate experiment (Experiment 2) that aimed to examine if the inclusion of an explicit context stimulus *in itself* led to a greater likelihood that stimuli would be remembered (as opposed to merely being recognized without any recall of associative detail), in the absence of any reward associations.

## Experiment 2

New subjects encountered trial-unique object stimuli where some trials included a task-irrelevant context picture in the background, and other trials lacked such an explicit context picture (i.e., context vs. no context conditions, manipulated on a within subject level for statistical efficiency). To prevent the context pictures from inadvertently producing any additional novelty effects, we pre-exposed subjects to the context pictures by having subjects perform a cover task (prior to encoding) that involved attending to focal object stimuli (not subject to memory test) with the task-irrelevant context pictures remained in the background. This pre-encoding-stage context exposure is similar to the learning stage of Experiment 1 in aiming to remove potential novelty effects on memory.

### Materials and Methods

#### Subjects

An additional 22 subjects were recruited for Experiment 2 (12 female, age range 18–34 years, mean age = 26.41, *SD* = 6.37). The procedures regarding recruitment, eligibility criteria, informed consent, and ethical clearance were identical to Experiment 1. To remove the any association that the object stimuli might have with reward, subjects were not able to explicitly win money on the task via their choices. However, to more generally motivate good concentration and participation, subjects were given a £2 bonus if their overall accuracy surpassed a certain threshold, in addition to the £6/h that they were paid as compensation for time.

#### Materials and Procedure

**FIGURE 4:**
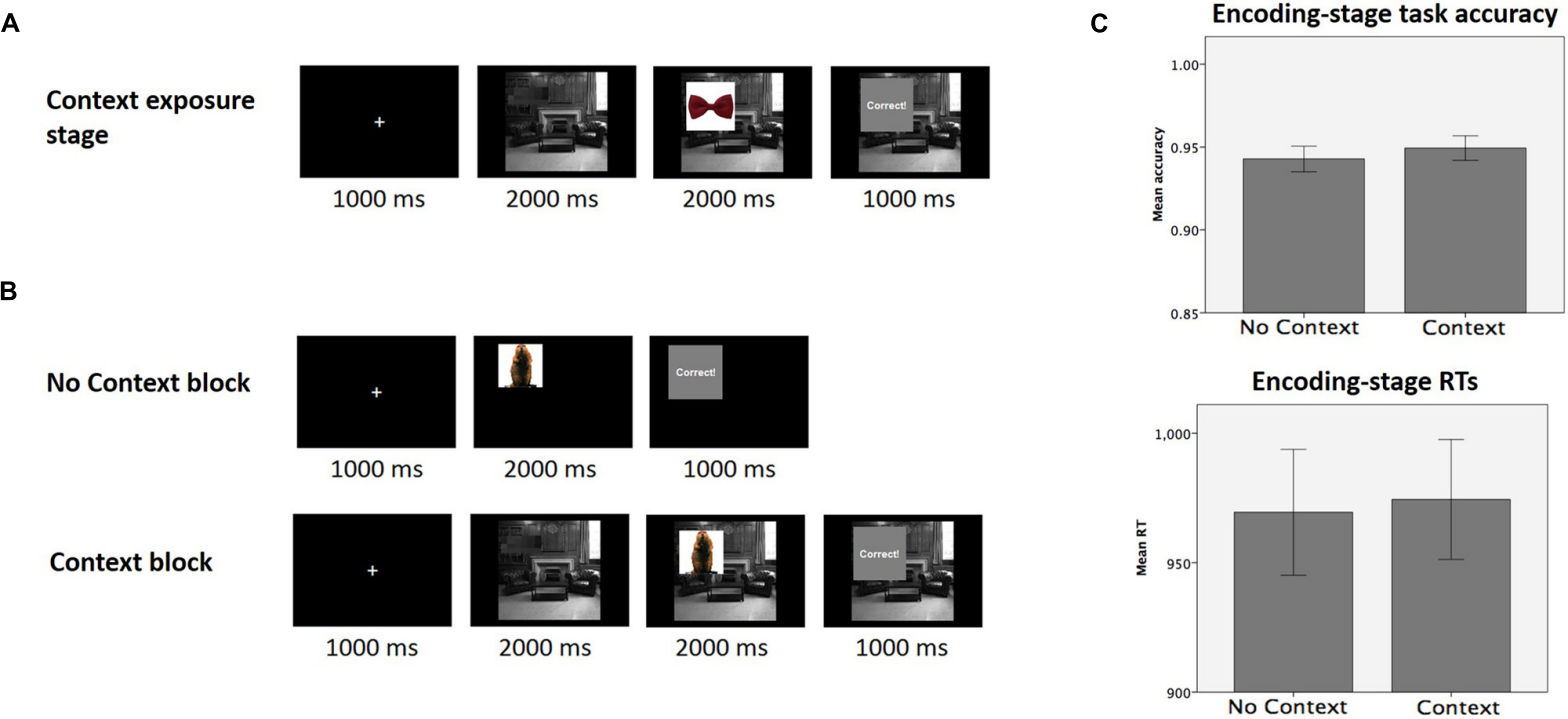
**Experiment 2 design and encoding-stage performance. (A)** Subjects were pre-exposed to the context pictures, in order to minimize the likelihood of incidental novelty-related effects in the encoding stage. Subjects completed a cover task in which they learned, via trial-and-error, which key presses (up/down) went with which object category, with the context pictures in the background **(B)** Trial sequence for the encoding-stage trials, for no context and context blocks (top and bottom, respectively; manipulated on a within-subject level). **(C)** Encoding-stage task accuracy and RTs.

Experiment 2 included three separate stages: a context exposure stage, encoding stage, and memory test. In Stage 1, the context exposure stage (**Figure 4A**), subjects performed a cover task in which they saw a series of objects, and made up- or down-arrow button presses in response to each object, with the context pictures in the background. For each object category, a certain response (up or down) was ‘correct’ 70% of the time, and subjects had to learn, via trial-and-error, which type of response went with which object category (Man-made or Natural) most of the time. On every trial, a context picture was shown by itself for 2000 ms before the object picture came onscreen (**Figure 4A**), and subjects were instructed that it was not relevant to the task that they had to perform. As in the previous experiment, the objects were randomly presented in any one of the four quadrants of the screen on each trial. Subjects were told whether their response was correct or wrong on every trial, but did not receive monetary reward for making correct responses. Subjects saw a total of 20 object stimuli and four context pictures in this stage (drawn from the pool of 16 unique context pictures in a similar manner as described in Experiment 1). The object and context pictures were repeated so that subjects completed a total of 64 trials in this stage of the experiment.

In Stage 2 (encoding stage, performed immediately after the context exposure stage), subjects completed a series of trials in which they saw pictures of objects (randomly presented in one of the four quadrants of the screen), and had to indicate if the objects were man-made or natural with left and right button presses (**Figure 4B**). On some trials, an obviously up-side down picture was shown, and subjects had to press the space-bar if such a picture came onscreen. Trials were divided into alternating ‘context’ and ‘no context’ blocks (three blocks in each condition, 44 trials in each block), and subjects were given the opportunity to take a break between each block. In ‘no context’ block trials, the object picture came onscreen immediately after the fixation-cross disappeared (**Figure 4B**). In the ‘context’ block trials, the fixation cross was followed by a context picture that was first presented on its own, and stayed onscreen for the rest of the trial. After the context picture was presented alone for 2000 ms, the object stimulus appeared superimposed on the context picture, and subjects then had to make the appropriate response. For both context and no context block trials, subjects received feedback regarding the accuracy of their responses on every trial, but were otherwise not rewarded for correct performance. Unlike the previous stage of the experiment, objects presented (240 in total, plus 24 up-side down targets) were trial-unique, while the context pictures were repeated, resulting in a total of 264 trials in this stage of the experiment. All stimuli used were identical to those used in Experiment 1.

In Stage 3, 5 days later, subjects returned to the lab to perform a surprise memory test, which proceeded identically as in Experiment 1.

### Results

Encoding-stage measures (task accuracy and RTs) and all memory measures were analyzed with a one-sample *t*-test comparing scores for the context versus the no context condition. The presence of an explicit context stimulus did not affect accuracy or RT during performance of the encoding-stage semantic categorization task [**Figure 4C**; accuracy: *t*(21) = 0.79, *p* > 0.4; RT: *t*(21) = 0.58, *p* > 0.5]. Memory scores were calculated identically as in Experiment 1, again including only objects that had received a correct response in the encoding-stage task.

**FIGURE 5:**
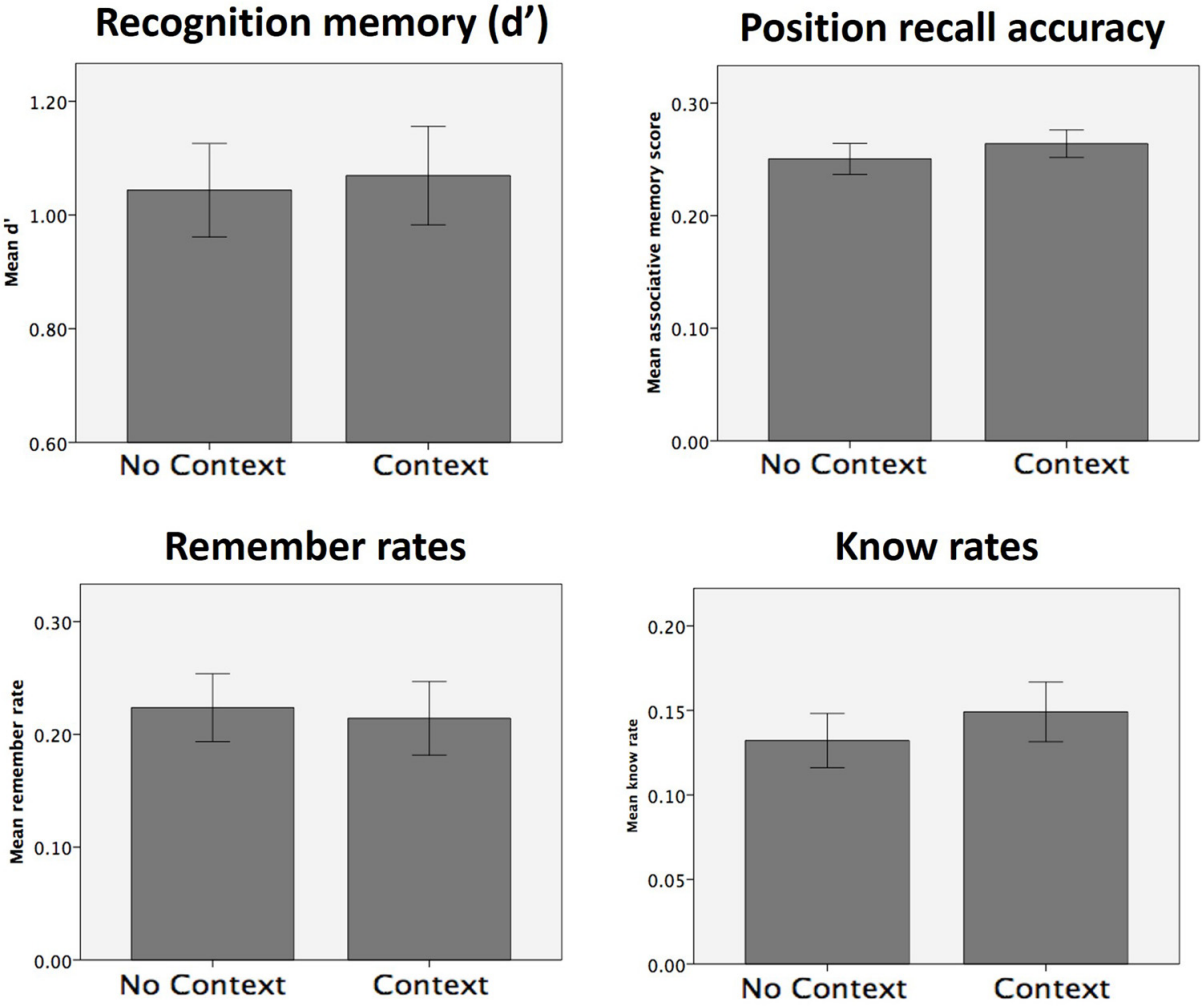
**Memory test results for Experiment 2.** No significant effects of context presence (vs. absence) were observed for any of the memory measures collected.

The presence of an explicit context stimulus did not have any significant effects on d′, position recall accuracy, remember rates or know rates (**Figure 5**; all *p* > 0.1; *p* > 0.27 using a one-tailed hypothesis for remember rates; see **Table 2** for all statistics). No significant context effects of were found on any of the other memory measures (sure hit rates, sure remember and sure know rates; all *p* > 0.1). In order to determine whether our data could be considered to show positive support in favor of the null hypothesis, we calculated the Bayes Factor for the null hypothesis that remember rates in the context and no context conditions are equivalent (Rouder et al., 2009). This analysis yielded a Bayes Factor of 43.35, which constitutes strong evidence for the null hypothesis that there is no difference between remember rates in the context and no context conditions (according to conventions wherein a Bayes Factor of 3.3 is typically considered ‘substantial’ evidence; Kass and Raftery, 1995).

Explicit comparisons of the effect of context on remember and know rates, using a repeated 2 × 2 ANOVA (context: context vs. no context; memory type: remember vs. know) found no significant effect of context [*F*(1,21) = 0.32, *p* > 0.5] or any context × memory type interaction [*F*(1,21) = 1.39, *p* > 0.2], though a main effect of memory type was found [*F*(1,21) = 4.33, *p* = 0.05], reflective of higher rates of remembering than knowing. Direct comparison of the remember > know rates between the two conditions also revealed no significant effects [i.e., (remember-context > know-context) > (remember-no context > know-no context); *p* > 0.2]. The Bayes Factor for this latter comparison was 17.79, which constitutes strong evidence in favor of the null hypothesis that the difference between remember and know rates was equivalent in the context and no context conditions. As such, this follow-up experiment found no evidence to suggest that the presence of an explicit context stimulus at encoding had any effect on remember rates or the balance between remember and know rates, compared to a relatively sparse context of a black background. Instead, Bayesian analysis indicates that the data presented here constitute strong evidence that the presence of an explicit context picture does not affect these memory measures.

## Discussion

In this study, we set out to examine if sharing a context with other rewarded events influenced memory for objects that were never associated with reward. We found that sharing a context with rewarding objects during encoding increased the probability that motivationally neutral objects would successfully recollected rather merely recognized, but only when the shared context was explicitly signaled with a background picture. Subjects who saw objects against a blank black background during encoding (no context condition) showed higher rates of remembering for rewarded compared to neutral objects, whereas subjects who saw a task-irrelevant context picture in the background during encoding (in the context condition) showed high rates of remembering for both rewarding *and* neutral objects (**Figure 2C**). These qualitative changes in memory were observed in the absence of any effects on overall recognition (as measured by d′; **Figure 2B**).

At memory test, subjects classified recognized objects as being having been ‘remembered’ or ‘known’ to be old, after having first made the initial judgment of whether they had seen the object before or not. Sharing a context with other rewarding objects did not affect simple recognition of neutral objects, but rather enhanced the likelihood that *associated detail* would be successfully recollected (resulting in a ‘remember’ rather than a ‘know’ memory categorization). This does not indicate that familiarity-based processes were necessarily absent on trials in which subjects made a ‘remember’ response. Rather, our results specifically indicate that memory for associated detail was more likely to be successfully recollected in the context condition (in response to being cued with the neutral objects), compared to in the no context condition. This high likelihood of successful recollection (following successful recognition) mirrored memory for the rewarding objects in both the context and no context conditions. Surprisingly, this improved recall of associative detail was not accompanied by an effect on the position recall scores (which serve as an objectively verifiable index of associative recall). This may have been due to position memory having been too low, resulting from the long delay between encoding and memory-test (memory test was conducted 5 days after encoding session; **Figure 2B**). Additionally, since subjects distinguish between ‘remember’ and ‘know’ memories on the basis of their ability to recall *any* associative detail (i.e., not specifically the position), it’s further highly plausible that their recollections related to associative detail other than the position (which was not a particularly salient feature of the encoding task). One possibility is that the context pictures were *themselves* recollected by subjects in the context condition (i.e., during the memory test; Experiment 1), leading to the enhanced rates of remembering for neutral objects in the context condition. To control for this possibility, we ran a second experiment (Experiment 2, outlined in **Figure 4**) to examine if the presence (versus absence) of a background picture during encoding would *itself* lead to better recollection. This follow-up experiment failed to find any evidence that the presence of a background context picture could itself lead to better recollection of associative detail (in support of better remembering) at subsequent memory test. Additionally, Bayesian analysis indicated that the results of this experiment constitute strong evidence in favor of the null hypothesis that the mere presence of a background context (relative to a blank black background) does not significantly recollection or change the balance between remembering and knowing in recognition. As such, it seems likely that the memory effects observed in Experiment 1 were not a result of the presence of the background context picture *per se*, but rather a result of the combination of the background picture and object-associated reward.

Another possibility is that neutral objects were indirectly reward conditioned via generalization of the reward association from the rewarded objects to the contexts, and then from the contexts to the neutral objects themselves. While we are unable to entirely rule out the possibility that reward associations may have spread in this manner, it seems unlikely given that our data shows no evidence of reward conditioning for either the contexts or the neutral objects themselves. The context pictures themselves were unlikely to have been reward-conditioned: because there was no relationship between the context pictures and the object’s reward status on each trial (a detail that was emphasized to subjects before the learning stage of the experiment), each context picture would have been paired with a rewarding objects roughly 50% of the time only. The lack of reward conditioning for the context pictures is further supported by the fact that, while reward significantly impacted both accuracy and RT in the encoding-stage task, the presence of a context picture itself did not significantly influence either RT or accuracy either alone or in interaction with reward (**Figure 2A**). As such, it seems unlikely that the observed memory effects would have come about via direct reward conditioning of the context pictures themselves.

What other neural mechanisms might allow sharing a context with separate rewarding objects to enhance memory for motivationally neutral objects? One possibility is that during encoding, both rewarding and neutral objects were bound to the same repeating context background pictures, so that representations of the neutral and rewarding objects were linked (along with that of the background context) at the level of the hippocampal ensemble. Multiple elements of an experience are thought to be bound together when they are encountered (Hayes et al., 2010; Boywitt and Meiser, 2012), and the hippocampus is thought to sub-serve this process via which multiple individual elements are bound into a single, conjunctive, long-term memory representation (O’Reilly and Rudy, 2001; Rudy and O’Reilly, 2001; Broadbent et al., 2002; Shimamura, 2002; Davachi, 2004). If the rewarding and neutral objects were indeed representationally linked in this context-mediated manner, reward-related mechanisms that support enhanced consolidation of rewarding events (e.g., dopamine release and synaptic tagging) may then have led to stabilization of the entire hippocampal engram, resulting in improved memory for the neutral objects as well. Spreading of reward-related memory enhancements to non-rewarded but associatively linked exemplars have been noted in the literature (Imai et al., 2014), and the findings reported here may be similar to such previous reports, pointing toward a similar phenomenon in which reward-related memory effects may spread to neutral objects that via indirect associative links.

Lastly, we also hypothesized that the similarity of the context pictures used might modulate context-mediated memory effects. Specifically, we had hypothesized that context-mediated memory effects might be more pronounced when the context pictures were similar compared to dissimilar, due to discrimination of the similar picture pair potentially placing a greater demand on hippocampal pattern separation (Marr, 1971; O’Keefe and Nadel, 1979; McNaughton and Morris, 1987; Graham et al., 2006; Kesner, 2007, 2013; Bakker et al., 2008; Bonnici et al., 2012; Mundy et al., 2013; Neunuebel and Knierim, 2014). However, we found no significant effects of context similarity on subsequent memory, either on its own or in interaction with reward (**Figure 3**). One limitation of these negative findings reported here are is that, because the context pictures were task irrelevant, some subjects in the context condition may not have successfully discriminated between the similar pictures at all. As such, we are unable to confidently assert, on the basis of the current data, that context similarity does not affect the observed context-mediated memory effects.

In this study, we set out to examine how extensively reward influences episodic memory. Specifically, we examined if sharing an explicit background context with separate rewarding objects influenced memory for objects that were themselves motivationally neutral. We found that sharing a context with rewarding objects during encoding increased the probability that neural objects would be successfully recollected as opposed to merely being recognized without any recall of associative detail, but only when the shared context was explicitly signaled with a background picture. These results indicate that reward-related effects on episodic memory may impact non-rewarded objects, possibly as a result of reward effects spreading through associative representational structures.

## Conflict of Interest Statement

The authors declare that the research was conducted in the absence of any commercial or financial relationships that could be construed as a potential conflict of interest.
